# Defining Core Evidence‐Based Practice Knowledge for Health Professionals: A Delphi Consensus Study

**DOI:** 10.1111/jep.70668

**Published:** 2026-10-15

**Authors:** Clara Carbonell‐Crespí, Albert Sesé‐Abad, Esther Díaz‐Mohedo, Juan Carlos Fernández‐Domínguez

**Affiliations:** ^1^ Ketty Ruiz Pelviperineal Physiotherapy Centre Palma Balearic Islands Spain; ^2^ Balearic Islands Health Research Institute (IdISBa) Palma Balearic Islands Spain; ^3^ Department of Psychology University of the Balearic Islands Palma Balearic Islands Spain; ^4^ Department of Physiotherapy University of Málaga Málaga Andalusia Spain; ^5^ Department of Nursing and Physiotherapy University of the Balearic Islands Palma Balearic Islands Spain

**Keywords:** clinical practice evaluation, content validity, Delphi technique, evidence‐based practice, health professions, knowledge assessment

## Abstract

**Rationale, Aims and Objectives:**

A clearly defined content domain is needed to assess whether health professionals possess the conceptual knowledge required for Evidence‐Based Practice (EBP). Existing competency frameworks and assessment instruments often combine knowledge with skills, attitudes and behaviours, limiting the identification of specific knowledge deficits. This study aimed to define the core EBP knowledge domain and obtain content validity evidence for a taxonomy applicable across health professions.

**Method:**

The study comprised three phases. First, two structured literature reviews identified relevant EBP competency frameworks and knowledge assessment instruments. These findings informed the operational definition of the EBP knowledge construct and the development of a preliminary seven‐dimension taxonomy. Content validity was then examined through a two‐round Delphi study. Eighteen experts participated in the first round and 17 in the second. Panellists rated the adequacy and relevance of each dimension and content area using a six‐point Likert scale and provided qualitative feedback. Agreement was defined as the proportion of experts assigning a score of 5 or 6, with a retention threshold of at least 80% for both criteria.

**Results:**

After the first Delphi round, several areas were reformulated, redistributed or removed to reduce conceptual overlap, and two dimensions were eliminated. In the second round, all retained dimensions and areas reached the agreement threshold except one newly introduced area, which was removed. The final taxonomy comprised 15 content areas organised into five dimensions: clinical question formulation, scientific literature search, critical appraisal, evidence implementation and knowledge translation, and evaluation of the EBP process.

**Conclusion:**

The taxonomy provides a consensus‐informed representation of core EBP knowledge and distinguishes conceptual knowledge from broader professional competence. It offers a basis for future objective assessment development and for identifying knowledge gaps in EBP training and implementation initiatives.

## 1 Introduction

Evidence‐Based Practice (EBP) has become a fundamental component of healthcare practice. This paradigm emphasises the integration of the best available research evidence, the clinical expertise of healthcare professionals, and patient values and preferences in clinical decision‐making [1]. The adoption of EBP has been associated with improved clinical outcomes, higher quality of care, and enhanced professional practice, as well as reduced variability in care and fewer clinical errors [2]. Moreover, EBP fosters a culture of continuous learning and professional development by requiring healthcare professionals to remain up to date with advances in scientific knowledge throughout their careers [3].

Despite its relevance, the integration of EBP into everyday clinical practice remains inconsistent across disciplines and healthcare organisations [4, 5, 6]. Previous studies have reported persistent gaps between what professionals are expected to do and what they are actually able to perform in terms of formulating clinical questions, searching for evidence, appraising studies, and integrating research findings into patient care [3, 7, 8]. These challenges highlight the need to strengthen the foundational knowledge that underpins the EBP process, both during academic training and across continuing professional development. Although implementation gaps are influenced by multiple individual, organisational, contextual, and discipline‐specific factors, including variation in how EBP competencies are defined, structured, and assessed across health professions [9, 10, 11, 12], foundational EBP knowledge remains a necessary component of effective evidence use.

From a knowledge translation perspective, several conceptual models emphasise the need to analyse barriers and facilitators at different levels of the healthcare system when promoting the adoption of evidence‐based practice [13, 14, 15]. Across these models, however, the ability of healthcare professionals to critically evaluate and apply scientific evidence presupposes the existence of a shared and sufficiently developed body of foundational EBP knowledge. When this core knowledge is insufficient or poorly defined, the subsequent stages of applying, implementing, and evaluating evidence in clinical practice may be compromised [8].

Effective engagement with EBP, therefore, requires more than exposure to research findings; it requires a coherent understanding of the principles, logic, and steps that structure the EBP process. This foundational knowledge enables professionals to judge the quality, relevance, and applicability of scientific evidence, particularly in clinical situations where explicit guidelines or best practice recommendations are unavailable.

Insufficient EBP knowledge and skills have been identified as important barriers to the adoption and implementation of evidence‐based practice [3, 8, 16]. Professionals with limited preparation may lack the conceptual and methodological tools needed to integrate evidence into clinical decision‐making. These findings highlight the need to strengthen foundational EBP preparation. However, there is limited consensus on what constitutes essential and transferable EBP knowledge. This lack of consensus complicates the assessment of professional preparedness, the identification of specific knowledge deficits, and the evaluation of training and implementation initiatives.

In parallel, numerous instruments have been developed to assess EBP‐related competencies and factors influencing their implementation. Systematic reviews of these instruments consistently identify limitations associated with their predominantly self‐reported nature, which undermines measurement objectivity and is vulnerable to bias and inaccurate self‐assessment [17, 18, 19]. Although objective assessment of EBP knowledge is critical for valid evaluation [9], relatively few tools directly measure this construct, and those that do are often profession‐specific, outdated, or difficult to implement at scale.

Existing EBP competency frameworks and assessment instruments do not consistently distinguish conceptual knowledge from procedural skills, attitudes and behaviours [9, 10, 20, 21]. This limits the interpretation of assessment results because an apparent deficit in overall EBP competence may reflect insufficient knowledge, limited practical skills, contextual barriers or a combination of these factors. A clearly defined representation of the EBP knowledge domain is therefore required before objective assessment instruments can be developed and used to evaluate professional preparedness, training outcomes and implementation initiatives [22, 23]. This is consistent with contemporary measurement frameworks, which emphasise that adequate content validity requires a clearly specified construct and content domain before other measurement properties can be meaningfully evaluated [24]. Subsequent psychometric evaluation should then examine the instrument's internal structure, including structural validity through approaches such as factor analysis, to determine whether theoretically distinct components are empirically supported [24, 25].

In this study, EBP knowledge is conceptualised as a distinct cognitive component of broader EBP competence. Whereas competence encompasses the integration of knowledge with procedural skills, attitudes, behaviours and professional performance, knowledge refers specifically to the conceptual understanding required to comprehend and engage with the EBP process.

To address these limitations, this study aimed to define the core EBP knowledge domain and obtain content validity evidence for a taxonomy intended for use across health professions. For the purposes of this study, “health professions” refers broadly to clinical disciplines involved in the delivery of healthcare, including medicine, nursing and allied health professions, whose practitioners are expected to engage with evidence‐based practice [26]. The taxonomy was developed to provide a conceptual basis for future objective assessment and to facilitate the identification of knowledge deficits that may otherwise remain obscured within broader measures of EBP competence.

## 2 Methods

The study was structured into three phases: Phase 0 involved two structured literature reviews; Phase 1 focused on developing the EBP knowledge taxonomy; and Phase 2 involved obtaining content validity evidence for the taxonomy through a Delphi study.

### 2.1 Phase 0: Structured Literature Reviews

Phase 0 comprised two structured literature reviews: the first examined taxonomies and theoretical frameworks for EBP competencies (step 1), and the second identified instruments designed to assess EBP knowledge among healthcare professionals (step 2).

Both reviews were conducted using multiple sources of scientific information in the health sciences, including databases of primary studies, systematic reviews, clinical practice guidelines, and additional scientific information sources. Searches for primary studies were performed in major biomedical and health sciences databases, including PubMed, CINAHL, IME, IBECS, LILACS, and Cuiden. Systematic reviews were identified through The Cochrane Library, PEDro, and the Joanna Briggs Institute. Clinical practice guidelines were retrieved from national and international guideline repositories, and additional sources were identified using EBSCOHost, BVS, Scopus, Web of Science, Trip Database, and SumSearch.

For the structured literature review on theoretical frameworks and taxonomies (step 1), search terms related to “evidence‐based practice,” “competencies,” “theoretical frameworks,” and “taxonomies” were used in English and Spanish, applying Boolean operators and truncation to maximize sensitivity. Eligible publications included theoretical frameworks and taxonomies specifically addressing EBP competencies among healthcare professionals, encompassing knowledge, skills, attitudes, and behaviours, and applicable across health disciplines. Frameworks were included if they provided a structured classification of competencies and had been applied or examined in empirical contexts. General implementation models not specifically focused on EBP competencies and frameworks lacking empirical support were excluded. The search prioritised publications in English or Spanish from the preceding 10 years. Earlier foundational EBP publications were also included when necessary to preserve conceptual continuity with the development of evidence‐based medicine and practice.

The second structured literature review (step 2) focused on instruments designed to measure EBP knowledge. Searches combined descriptors related to “knowledge,” “evidence‐based practice,” and “questionnaires” in English and Spanish, using Boolean operators, truncation, and synonyms to capture a wide range of instruments. Studies were included if they reported the development of instruments intended to assess EBP knowledge in healthcare professionals from any discipline and if at least one psychometric or measurement property had been evaluated [24, 25, 27]. Instruments that did not directly assess knowledge, such as those focused exclusively on attitudes, barriers, or facilitators, were excluded. The same language and temporal criteria were applied as in the first review.

The identified instruments were examined with respect to their reported dimensional structure, measurement properties, assessment format, and alignment with the theoretical frameworks and taxonomies identified in step 1.

The initial searches were conducted by the principal investigator and subsequently updated by another member of the research team. Article selection, data extraction, and evaluation of the identified instruments were performed independently by both investigators, and disagreements were resolved through discussion until consensus was reached.

### 2.2 Phase 1: Development of the EBP Knowledge Taxonomy

Phase 1 of the study took place between January 2023 and February 2024, with the aim of defining the EBP knowledge construct and developing a preliminary taxonomy of the core conceptual knowledge required across health professions.

The findings from the two structured literature reviews were synthesised to develop an operational definition of the EBP knowledge construct and identify the principal content domains represented in existing frameworks and assessment instruments. The principal investigator proposed an initial taxonomy model, which was then reviewed and discussed with two additional researchers until a preliminary consensus‐based version was reached.

Through iterative meetings, the research team reviewed the definition, scope, organisation, and wording of the proposed dimensions and content areas. Revisions were made to reduce conceptual overlap, distinguish knowledge from skills and attitudes, and ensure consistency with the evidence identified in Phase 0. The resulting preliminary taxonomy was subsequently submitted to the expert panel in Phase 2.

### 2.3 Phase 2: Delphi Study for Obtaining Content Validity Evidence for the EBP Knowledge Taxonomy

#### 2.3.1 Design

This study employed a two‐round Delphi design in accordance with the CREDES guidelines [28]. The study was conducted from February to June 2024.

#### 2.3.2 Participants

A purposive panel of 18 experts in evidence‐based practice (EBP) was invited to participate in the Delphi study. Expert selection was based on academic, methodological, research, educational, and implementation‐related expertise in EBP and health sciences, rather than on professional category alone.

Expertise was assessed according to academic qualifications, research experience, professional trajectory, and documented involvement in EBP methodology, clinical research, psychometrics, evidence synthesis, health sciences education, or implementation of evidence‐informed practice. Seventeen panellists held a doctoral degree (94.4%), and one held a master's degree in Epidemiology together with extensive professional and research experience. In terms of disciplinary background, 12 panellists were from nursing, 5 from physiotherapy, and 1 from psychology. These disciplines fall within the broad definition of health professions adopted in this study, encompassing nursing and allied health professions whose practitioners are expected to engage with evidence‐based practice. Although the panel did not include representatives from every discipline encompassed by this broader definition, experts were selected primarily for their demonstrated expertise in EBP and related methodological domains rather than to achieve proportional representation across professions.

In line with the considerations proposed by Baker et al. for defining expertise in Delphi studies [29], panellists were selected on the basis of demonstrable knowledge and experience relevant to the study topic. Eligible experts were required to have published at least one EBP‐related article in a Journal Citation Reports‐indexed journal or to have participated in EBP research, clinical practice protocols, or evidence‐informed guidelines. They were also required to meet at least one additional criterion: an advanced postgraduate qualification; publication experience in EBP or health sciences methodology; participation in EBP‐related research, guidelines, protocols, or educational initiatives; or an academic, research, clinical, or managerial role related to the promotion or application of EBP.

The panel was composed of experts with complementary academic, methodological, clinical, educational, and managerial profiles, including university academics, senior lecturers, full professors, researchers, practising clinicians, clinical managers, and professionals involved in teaching, promoting, or implementing EBP in healthcare contexts.

### 2.4 Ethical Considerations

According to applicable institutional and national regulations, formal research ethics committee approval was not required for this anonymous, minimal‐risk expert consultation. All procedures were conducted in accordance with the principles of the Declaration of Helsinki.

All invited experts received an email with an information letter describing the aims of the study, the Delphi procedure (number of rounds, type of questions and rating scales), the approximate time required to complete each round, the voluntary nature of participation, the anonymous handling of responses and how quantitative ratings and qualitative comments would be used. The introductory text of the online questionnaire reiterated the objective of the study and explained how the data would be used. By choosing to access and complete the online questionnaire after reading this information, panellists provided informed consent to participate. No directly identifying information was collected, responses were recorded anonymously, and participants could discontinue the survey at any time by closing the form.

### 2.5 Procedure

The experts were initially contacted by email and provided with an explanatory video and a diagram of the preliminary taxonomy. Each dimension and content area was evaluated according to two criteria: adequacy, defined as its appropriateness and consistency with the EBP knowledge construct; and relevance, defined as its importance for representing the content domain. Data were collected through an online questionnaire administered using Google Forms.

A six‐point Likert scale ranging from 1 (*strongly disagree*) to 6 (*strongly agree*) was used for both criteria. When a score below 5 was assigned, panellists were asked to justify their rating and, where appropriate, propose an alternative wording or modification. For each dimension and content area, agreement was calculated separately for adequacy and relevance as the proportion of panellists assigning a score of 5 or 6. The prespecified consensus threshold was at least 80% agreement for both criteria [30]. Dimensions or content areas that did not meet this threshold were reviewed in conjunction with the qualitative comments and were reformulated, redistributed, or removed, as appropriate.

Qualitative comments were reviewed by the three‐member research team and grouped according to the dimension or content area to which they referred. Proposed changes were discussed until agreement was reached, and only modifications consistent with the construct definition and quantitative consensus results were incorporated into the revised taxonomy.

All 18 experts completed the first Delphi round. For the second round, panellists received the revised taxonomy together with a summary of the changes made in response to the first‐round ratings and comments. Seventeen experts completed the second round; one panellist withdrew for personal reasons. The same rating criteria, response scale, and consensus threshold were applied in both rounds.

## 3 Results

### 3.1 Phase 0: Structured Literature Reviews (Steps 1 and 2)

As a result of step 1, the following theoretical frameworks and taxonomies for assessing EBP competencies were identified: Sackett's five‐step model [31, 32], the Sicily Statement on Evidence‐Based Practice [20], the CanMEDS Competency Framework [33], the Evidence‐Based Practice Competencies for RNs and APNs [21], the Competency Framework for EBP in Nursing [34], and the Core Competencies in EBP for Health Professionals [11] (Table 1).

**Table 1:** Description of the main characteristics of theoretical frameworks and competence taxonomies in EBP.

Theoretical framework/Taxonomy	Description	Year	Country	Relevance to EBP knowledge assessment
Taxonomy of the five steps of EBP (Evidence‐Based Medicine Working Group, 1992; Sackett et al., 1996)	Initial 5‐step EBP model (Ask, Acquire, Appraise, Apply, Assess)	1992–1996	Canada	Basis for measuring sequential EBP knowledge; useful for step‐focused testing.
Sicily Statement on Evidence‐Based Practice (Dawes et al., 2005)	International consensus detailing theoretical and practical competences for the same five steps, with a formative approach and adaptable to different levels of training.	2005	Italy	Allows for the development of assessment tools focusing on conceptual and practical understanding of each phase of the process, from ‘asking’ to ‘measuring results’.
CanMEDS Competency Framework (Frank & Danoff, 2007)	It proposes 7 Practitioner Roles (e.g., Scholar, Communicator, Collaborator), where the search for and interpretation of evidence for decision‐making is integrated.	2007	Canada	The assessment of the cognitive component of EBP is mainly linked to the Scholar role, useful to measure the understanding and use of evidence in clinical practice.
Evidence‐Based Practice Competencies for RNs/APNs (Melnyk et al., 2014)	Specific competencies for nurses: 13 for RNs and an additional 11 for APNs, describing EBP theory and practice.	2014	United States	Assesses essential knowledge (e.g., basic statistics, bibliographic resources) and its practical translation (leading evidence implementation) with a specific focus on nursing.
Competency Framework for EBP in Nursing (Leung et al., 2016)	Based on the 5‐step model, it separates attributes of knowledge and practical skills within nursing.	2016	Australia	Facilitates differentiated assessment of theoretical understanding (what the professional knows about searching, critical reading) and practical implementation of EBP in clinical nursing settings.
Core Competencies in EBP (Albarqouni et al., 2018)	It defines 68 competencies derived from a systematic review and an international Delphi, applicable to multiple professions and at different levels of depth.	2018	International	Helps to measure theoretical knowledge and its different degrees (mentioned, explained, practiced) to adapt the assessment to various professional contexts, assessing the understanding and use of evidence.

Abbreviations: APNs, Advanced Practice Nurse; RNs, Registered Nurse.

The identified frameworks described EBP as a multidimensional process encompassing combinations of knowledge, skills, attitudes, behaviours, and professional performance. The five‐step model and the Sicily Statement primarily organised the methodological stages of EBP, whereas CanMEDS and the nursing‐specific competency frameworks incorporated both cognitive and procedural components. The multiprofessional framework identified by Albarqouni et al. also formulated competencies in terms of what health professionals should know and be able to perform. None of the identified frameworks presented conceptual EBP knowledge as a fully independent content domain.

As a result of step 2, eight instruments designed to assess EBP knowledge or broader EBP‐related constructs were identified: the Fresno Test and its adaptations [35, 36, 37, 38, 39], the Evidence‐Based Practice Questionnaire [40] (EBPQ), the Evidence‐Based Practice Knowledge Assessment in Nursing [41] (EKAN), the Evidence‐Based Practice Knowledge, Attitudes, and Behaviour Questionnaire [42] (EBP‐KABQ), the Evidence‐Based Practice Competence Questionnaire [43] (EBP‐COQ), the Developing Evidence‐Based Practice Questionnaire [44] (DEBP), the EBE‐Student questionnaire [45], and the Evidence‐Based Medicine Questionnaire [46] (EBMQ) (Table 2).

**Table 2:** Main Characteristics of instruments assessing EBP knowledge and broader EBP competencies.

Instrument	Year	Country	Target population	Constructs assessed	Assessment format	Scope
Fresno Test	2003	United States	Physicians and medical trainees; later adapted to other health professions	Applied EBP knowledge and skills, including question formulation, searching, appraisal, and quantitative interpretation	Performance‐based, open‐ended clinical scenarios scored using standardised rubrics	Originally developed for medicine; subsequently adapted to several health professions
EBPQ	2006	United Kingdom	Nurses; later used across health professions	EBP practice/use, attitudes, and self‐reported knowledge and skills	Self‐report questionnaire	Initially nursing; broader subsequent use
EKAN	2015	United States	Nurses and nursing students	Objective EBP knowledge	Objective item‐based assessment evaluated using Rasch modelling	Nursing‐specific
DEBP	2007	United Kingdom	Nurses	Sources of knowledge, barriers, facilitation and support, and self‐reported EBP skills	Self‐report questionnaire	Nursing‐specific; later adaptations
EBP‐KABQ	2003	Hong Kong	Undergraduate medical students	EBP knowledge, attitudes, and behaviour	Questionnaire combining knowledge items with self‐reported attitudinal and behavioural components	Initially undergraduate medicine; later modified across professions
EBP‐COQ	2013	Spain	Undergraduate nursing students	Attitudes, knowledge and skills, and EBP application/use	Self‐report questionnaire	Initially nursing students
EBE‐ST	2021	Spain	Undergraduate nursing students	Evidence searching, interpretation and use, education, clinical application, and professional perspectives	Self‐report questionnaire	Nursing students
EBMQ	2018	Malaysia	Primary care physicians	EBM knowledge, practice, barriers, access, patient preferences, and support	Mixed factual and self‐report questionnaire	Primary care medicine

Among the identified instruments, the EKAN directly assessed EBP knowledge using an objective response format, whereas the Fresno Test and its adaptations used performance‐based clinical scenarios to assess applied EBP knowledge and skills. The remaining instruments were predominantly self‐reported and commonly assessed several constructs, including knowledge, attitudes, skills, behaviors, self‐efficacy, or contextual factors. The EKAN was developed specifically for nursing, while the Fresno Test has been adapted for different health professions and cultural contexts. However, its open‐ended format requires trained assessors and substantial scoring resources, which may limit its feasibility for large‐scale administration.

Table 3 summarises the reported dimensional structure and measurement properties of the identified instruments and describes their conceptual alignment with the EBP knowledge construct.

**Table 3:** Reported dimensional structure, measurement properties, and conceptual alignment of instruments assessing EBP knowledge and broader EBP competencies.

Instrument	Reported dimensional structure	Main measurement evidence reported	Relationship with EBP knowledge construct
Fresno Test	No latent dimensional structure established in the original study; performance represented primarily by a total competence score across open‐ended items	Internal consistency, inter‐rater reliability, item discrimination, known‐groups validity, and responsiveness were reported across the original study and subsequent adaptations	Assesses applied EBP knowledge and skills and does not isolate conceptual knowledge from performance
EBPQ	Three factors: EBP practice, attitudes towards EBP, and self‐reported knowledge and skills	Factor‐analytic support, internal consistency, convergent and discriminant validity; subsequent studies examined confirmatory fit and invariance	Includes self‐reported knowledge and skills but does not isolate objective conceptual knowledge
EKAN	Objective item‐based assessment examined using Rasch analysis rather than a conventional multidimensional subscale model	Rasch‐based item functioning, reliability, and validity evidence reported	Directly targets objective EBP knowledge but was developed specifically for nursing
DEBP	Multidimensional structure addressing sources of knowledge, barriers, and organisational support or facilitation	Development and reliability evidence reported; focused mainly on influences on EBP development	Assesses implementation‐related factors and self‐perceived skills rather than objective conceptual knowledge
EBP‐KABQ	Combined assessment of knowledge, attitudes, and behaviour; no independent latent knowledge domain established	Development and validation evidence reported for the combined questionnaire	Knowledge‐related items are embedded within broader attitudinal and behavioural assessment
EBP‐COQ	Three factors in students: attitudes, knowledge and skills, and application; four domains in the professional version: attitudes, knowledge, skills, and utilisation	Factor‐analytic support and internal consistency reported; professional version also included confirmatory analyses	Includes self‐perceived knowledge but does not objectively or fully separate knowledge from skills
EBE‐ST	Eight‐factor self‐report structure concerning EBP education, evidence searching and interpretation, clinical use, and professional application	Exploratory psychometric evidence and internal consistency reported	Assesses perceptions and educational experiences rather than objective conceptual knowledge
EBMQ	Eight‐domain structure including knowledge, practice, access, barriers, patient preferences, and support	Content and construct validity and reliability evidence reported	Includes factual knowledge but combines it with practice, barriers, and contextual domains

### 3.2 Phase 1: Development of the Preliminary EBP Knowledge Taxonomy

The cognitive components represented in the frameworks and instruments identified in Phase 0 were synthesised to distinguish conceptual EBP knowledge from procedural skills, attitudes, and behaviours.

The EBP knowledge construct was operationally defined as the structured body of conceptual knowledge that health professionals require to understand and engage with the EBP process. It encompassed knowledge of the principles and rationale underlying clinical question formulation, evidence retrieval, critical appraisal, implementation, and evaluation. It also included relevant methodological and statistical concepts and an understanding of how research evidence is integrated with clinical expertise and patient values and preferences. Procedural skills, attitudes, and self‐reported behaviours were excluded from the construct. Accordingly, EBP knowledge was treated as one component of broader EBP competence rather than as synonymous with competence itself.

Based on this operational definition, a preliminary taxonomy comprising seven dimensions was developed (Supplemental Information A).

The preliminary taxonomy was informed by the classical EBP process described in foundational publications [1, 20, 31] and by later competency frameworks distinguishing, to varying degrees, knowledge from applied skills and professional performance [11, 21].

The seven preliminary dimensions are summarised below and presented in Figure 1.

**Figure 1:**
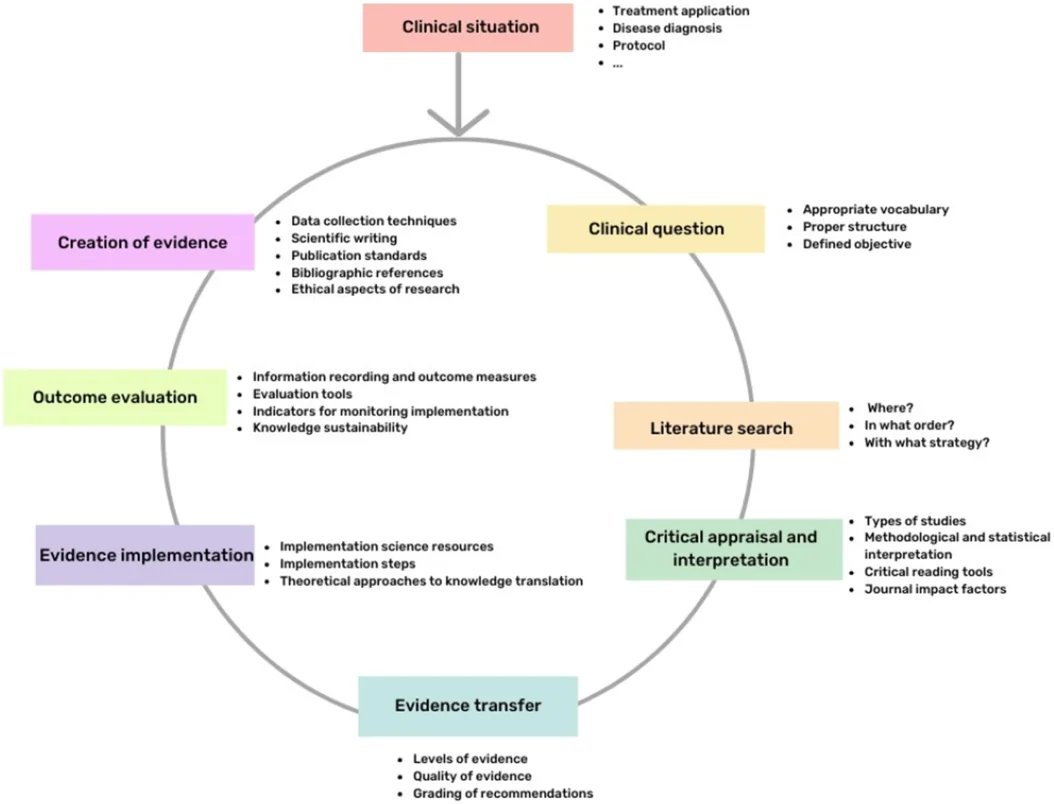
Preliminary seven‐dimension EBP knowledge taxonomy submitted to the Delphi panel.

Dimension 1, Clinical Question Formulation, comprised three content areas: translating natural language into scientific language, structuring the question, and determining its objective.

Dimension 2, Scientific Literature Search, comprised three content areas: electronic resources for evidence searching, the order of the external evidence search, and search strategy development.

Dimension 3, Critical Appraisal of Results, comprised four content areas: study types, statistical interpretation, critical appraisal tools, and journal impact indicators.

Dimension 4, Classification and Hierarchy of Scientific Evidence, comprised three content areas: levels of evidence, quality of evidence, and grades of recommendation.

Dimension 5, Evidence Implementation and Knowledge Translation, comprised three content areas: theoretical approaches to knowledge translation, implementation science resources, and implementation stages.

Dimension 6, Evaluation of the EBP Process, comprised four content areas: documentation and outcome measures, instruments for analysing information, implementation evaluation indicators, and sustainability of knowledge use.

Dimension 7, Generation of Scientific Evidence, comprised five content areas: data collection techniques, scientific writing, reporting standards, bibliographic references, and research ethics.

### 3.3 Phase 2: Delphi Study

The progression of the taxonomy through the two Delphi rounds is presented in Figure 2.

**Figure 2:**
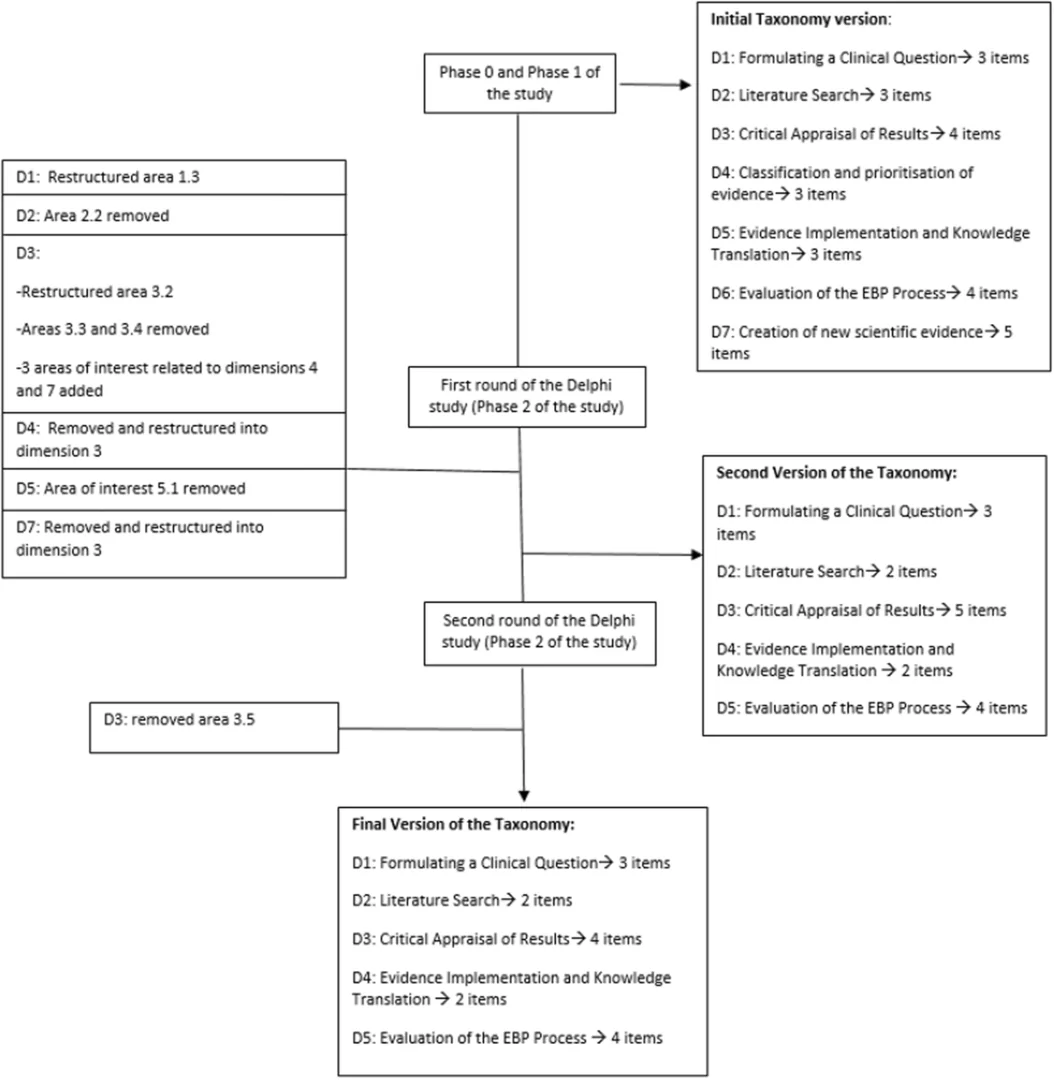
Flowchart of the Development of the EBP knowledge taxonomy across the two Delphi rounds.

### 3.4 First Delphi Round

All 18 invited experts completed the first Delphi round. Seventeen held a doctoral degree (94.4%), and one held a master's degree in Epidemiology combined with extensive professional and research experience. Fourteen panellists (77.8%) had documented expertise in EBP, evidence‐informed practice, clinical practice guidelines, or implementation, and 15 (83.3%) had expertise in health sciences methodology, clinical research, epidemiology, evidence synthesis, psychometrics, systematic reviews, or measurement‐related research. Six panellists (33.3%) also held postgraduate qualifications in complementary methodological, educational, managerial, or health‐related fields.

Panellists were based in different geographical areas of Spain, including Andalusia, Murcia, the Balearic Islands, Madrid, the Basque Country, and Catalonia.

Agreement levels for the adequacy and relevance of the preliminary dimensions and content areas are presented in Table 4. All content areas within Dimension 1 reached the prespecified threshold, although minor wording changes were introduced. In Dimension 2, one content area was removed because of conceptual redundancy. Within Dimension 3, area 3.4 did not reach the agreement threshold and was removed. Area 3.3 was also removed following consideration of qualitative feedback and conceptual overlap, while area 3.2 was reformulated and content from Dimensions 4 and 7 was incorporated into the revised dimension.

**Table 4:** Percentage agreement from the first round of the Delphi study on the dimensions and content areas of the proposed EBP knowledge taxonomy.

	% Agreement	
Dimension or content area	Adequacy/Pertinence	Relevance
**Dimension 1: Formulation of clinical question(s) regarding practice**	100	100
1.1: Translating natural language into scientific language	88.8	94.4
1.2 Structure the question	83.3	94.4
1.3 Determine the objective	88.8	88.8
**Dimension 2: Scientific literature search**	88.8	100
2.1 Electronic resources for evidence search	100	83.3
2.2 Search for external evidence: order of search	88.8	88.8
2.3 Search strategy	88.8	88.8
**Dimension 3: Critical appraisal of results**	94.4	88.8
3.1 Study Types	94.4	94.4
3.2 Statistical Interpretation of the Study	88.8	94.4
3.3 Critical Reading Tools	94.4	88.8
3.4 Journal Impact Indices	27.7	22.2
**Dimension 4: Classification and hierarchy of scientific evidence**	88.8	88.8
4.1 Levels of Evidence	83.3	83.3
4.2 Quality of evidence	77.7	83.3
4.3 Grades of Recommendation	88.8	88.8
**Dimension 5: Implementation of Evidence and Knowledge Translation**	88.8	94.4
5.1 Theoretical Approaches to Knowledge Translation	77.7	77.7
5.2 Implementation Science Resources	83.3	72.2
5.3 Implementation Steps	94.4	94.4
**Dimension 6: Evaluation of the EBP process**	94.4	88.8
6.1 Recording of Information and Outcome Measures	94.4	94.4
6.2 Tools or Instruments for Analysing Information	77.7	77.7
6.3 Indicators for implementation evaluation	94.4	94.4
6.4 Sustainability of Knowledge Utilisation	94.4	94.4
**Dimension 7: Generation of new scientific evidence**	66.6	66.6
7.1 Data collection techniques	72.2	72.2
7.2 Scientific Writing	55.5	61.1
7.3 Publication Standards for Studies	55.5	61.1
7.4 Bibliographic References	66.6	66.6
7.5 Ethical Aspects of Research	77.7	77.7

Dimension 4 was removed because its content overlapped with the critical appraisal dimension, and the relevant concepts were redistributed. Within Dimension 5, two content areas did not meet the agreement threshold; one was removed, while the remaining content was reconsidered and reorganised in the revised taxonomy. Within Dimension 6, one content area did not meet the agreement threshold and was reformulated following qualitative feedback. Dimension 7 was removed as an independent dimension; one of its content areas was retained and reassigned to another dimension.

### 3.5 Second Delphi Round

Following revision of the taxonomy after the first round (Supplemental Information B), 17 experts completed the second round; one panellist withdrew for personal reasons. All retained dimensions and content areas reached the prespecified 80% agreement threshold for both adequacy and relevance, except the newly introduced area 3.5, “Bibliographic References”, which obtained 64.7% agreement and was removed.

The final taxonomy comprised 15 content areas organised into five dimensions: Clinical Question Formulation (three areas), Scientific Literature Search (two areas), Critical Appraisal of Results (four areas), Evidence Implementation and Knowledge Translation (two areas), and Evaluation of the EBP Process (four areas). The final taxonomy is presented in Supplemental Information C (Table 5).

**Table 5:** Percentage agreement from the second round of the Delphi study on the dimensions and content areas of the proposed EBP knowledge taxonomy.

	% Agreement	
Dimension or content area	Adequacy/Pertinence	Relevance
**Dimension 1: Formulation of a clinical question**	100	100
1.1: Translating natural language into scientific language	100	100
1.2 Structure the question	88.2	100
1.3 Types of clinical questions	94.1	94.1
**Dimension 2: Scientific literature search**	100	100
2.1 Electronic resources and sources of evidence	94.1	94.1
2.2 Search strategy	100	94.1
**Dimension 3: Critical appraisal of results**	100	100
3.1 Study Types	94.1	100
3.2 Methodological appraisal	100	100
3.3 Applicability of the Evidence	94.1	88.2
3.4 Ethical Aspects of Research	94.1	94.1
3.5 Bibliographic References	64.7	64.7
**Dimension 4: Implementation of Evidence and Knowledge Translation**	100	100
4.1 Implementation Science Resources	88.2	94.1
4.2 Implementation Steps	100	94.1
**Dimension 5: Evaluation of the EBP process**	94.1	94.1
5.1 Recording of Information and Outcome Measures	100	94.1
5.2 Tools or Instruments for Analysing Information	88.2	88.2
5.3 Indicators for implementation evaluation	94.1	94.1
5.4 Sustainability of Knowledge Utilisation	94.1	94.1

## 4 Discussion

The results of the Delphi process provide both quantitative and qualitative evidence regarding the content and conceptual coherence of the developed taxonomy. In the first round, 100% agreement was observed in dimensions such as “Formulation of the Clinical Question” and “Scientific Literature Search,” underscoring the relevance and clarity of these components within the proposed EBP knowledge domain. In Dimension 3 (Critical Appraisal of the Results), the “Journal Impact Indices” area was eliminated due to its perceived low relevance, highlighting the need to focus the taxonomy on aspects that unequivocally reflect conceptual knowledge, while avoiding elements that may lead to errors or an overestimation of practical competencies [17, 19].

Adjustments were also made to incorporate aspects such as the analysis of qualitative designs, which were identified as relevant by some experts. Moreover, Dimension 7, “Generation of New Scientific Evidence,” received lower agreement and was therefore eliminated as an independent component. Nevertheless, areas such as “Ethical Aspects of Research” were relocated to other, more relevant dimensions. This iterative refinement strengthened the conceptual coherence of the taxonomy and improved its consistency with the operational definition of EBP knowledge [47, 48].

In the second Delphi round, both the adequacy and relevance agreement percentages reached the minimum prespecified threshold across all dimensions and content areas, except for the newly introduced area “Bibliographic References,” which was removed. These findings provided further content validity evidence for the revised taxonomy. Beyond the numerical consensus, experts provided qualitative feedback emphasising that an explicit theoretical framework is essential for identifying educational gaps that may be masked by global competence assessments.

Traditional theoretical frameworks, such as the 5‐Step Model by Sackett et al. and the Sicily Statement on Evidence‐Based Practice, have laid the foundation for EBP [1, 20]. These models postulate the competencies a good practitioner should possess, considering both theoretical knowledge and practical skills. This has paved the way for the development of more recent competency frameworks [3, 9, 11, 21, 26] as well as various EBP assessment tools, some with greater methodological rigour than others [9, 10, 25, 44, 46]. However, most of these models conceptualise EBP globally, intertwining knowledge, skills, and attitudes without explicitly delineating the theoretical component. This raises important questions regarding the precision of assessment and the ability to detect specific educational needs.

By isolating the theoretical aspect, this taxonomy seeks to complement existing proposals and provide a solid foundation for exclusively evaluating knowledge. Clearly defining the construct of “EBP knowledge” establishes a precise nomenclature from the outset, thus avoiding the common confusion between “competence” and “knowledge” [9, 11, 17, 19, 21]. Knowledge should therefore be distinguished from competence, as it constitutes an essential component of professional competence but does not encompass competence in its entirety. Professional competence also requires the application and adaptation of knowledge and skills to specific clinical contexts, together with context‐dependent attitudes and professional attributes [49]. Accordingly, assessing EBP knowledge captures an important cognitive foundation of competence but should not be interpreted as an assessment of overall EBP competence, which encompasses the integration and application of knowledge within professional practice. This distinction may improve the detection of conceptual gaps and support the creation of more specific and objective evaluative instruments. Core EBP knowledge should be understood as a necessary but not sufficient condition for implementation. Barriers such as time constraints, organisational culture, access to resources, leadership support, and professional role expectations may also influence whether evidence is translated into practice.

From a practical perspective, this taxonomy may be integrated into undergraduate and postgraduate health sciences training programs. In doing so, it could support the prioritisation of fundamental areas, such as the formulation of clinical questions (Dimension 1) and the planning of systematic, exhaustive searches in the scientific literature (Dimension 2), helping healthcare professionals develop a solid foundation in EBP from the early stages of their careers [3, 50]. Moreover, its implementation could help identify specific knowledge gaps, such as in the critical appraisal of scientific evidence (Dimension 3) or in the implementation and monitoring of evidence‐based interventions (now reorganised in the revised taxonomy), thereby providing a conceptual reference for designing tailored educational interventions and knowledge transfer strategies suited to the particularities of each setting [16].

The taxonomy also incorporates the need to understand applicability, including whether evidence is relevant to a specific patient, clinical context, available resources, and patient preferences. This distinction is essential because critical appraisal does not end with methodological quality; it also requires judging whether evidence can reasonably inform decision‐making in a given care context.

Additionally, the systematic perspective introduced in Dimension 5 emphasises the relevance of recording outcomes and analysing information, linking conceptually to monitoring the impact of EBP on patient care. In contrast, aspects more closely related to broad implementation outcomes may be more relevant to advanced or leadership roles, suggesting the taxonomy may inform differentiated learning pathways.

Finally, the results of this study have potential implications for clinical practice. By addressing the inherent limitations of self‐reported questionnaires, such as social desirability bias [17, 19], this taxonomy provides a conceptual basis for the development of objective, reproducible assessment tools that could potentially be used across various health professions. Future research should focus on operationalising this taxonomy into item banks and evaluating their psychometric properties, as well as exploring integration with multidimensional tools such as the HS‐EBP questionnaire [51] to support more precise evaluation of EBP knowledge and broader EBP‐related competence.

### 4.1 Limitations

This study has several limitations. First, the Delphi panel was composed of experts from a single country and was primarily represented by nursing, with additional participation from physiotherapy and psychology. Although expert selection was based on demonstrated EBP and methodological expertise rather than proportional representation across professions, future validation studies could include a broader range of disciplinary and international perspectives. Second, expert selection may have introduced selection bias, as participants were recruited according to predefined expertise criteria. Third, the taxonomy provides conceptual and content validity evidence but has not yet been empirically tested through item development or psychometric analysis. Finally, the structured literature reviews prioritised publications from the preceding 10 years, which may have excluded earlier influential work in evidence‐based medicine, although foundational references were considered in the conceptual development of the taxonomy.

## 5 Conclusion

This study has provided content validity evidence for a taxonomy of essential knowledge for EBP among healthcare professionals. The adjustments made throughout the Delphi process, along with the high level of agreement achieved in the Delphi study, underscore the relevance and conceptual coherence of this proposal. The taxonomy clarifies what constitutes core EBP knowledge, distinguishes it from other components of competence, and may support curriculum design, assessment, and educational planning.

This taxonomy represents a structured conceptual basis for the development of an objective knowledge assessment tool in this area. Further international consultation and psychometric evaluation are required before its applicability across health professions and settings can be established.

## Author Contributions

Conceptualisation: Clara Carbonell‐Crespí, Albert Sesé‐Abad and Juan Carlos Fernández‐Domínguez. Methodology: Clara Carbonell‐Crespí, Albert Sesé‐Abad and Juan Carlos Fernández‐Domínguez. Formal analysis: Clara Carbonell‐Crespí, Albert Sesé‐Abad and Esther Díaz‐Mohedo. Investigation: Clara Carbonell‐Crespí, Albert Sesé‐Abad and Esther Díaz‐Mohedo. Data curation: Clara Carbonell‐Crespí. Validation: Clara Carbonell‐Crespí, Albert Sesé‐Abad, Esther Díaz‐Mohedo and Juan Carlos Fernández‐Domínguez. Visualisation: Esther Díaz‐Mohedo. Writing – original draft: Clara Carbonell‐Crespí, Juan Carlos Fernández‐Domínguez and Albert Sesé‐Abad. Writing – review and editing: Clara Carbonell‐Crespí, Albert Sesé‐Abad, Esther Díaz‐Mohedo and Juan Carlos Fernández‐Domínguez. Supervision: Albert Sesé‐Abad and Juan Carlos Fernández‐Domínguez. All authors approved the final version of the manuscript and agree to be accountable for all aspects of the work.

## Funding

The authors have nothing to report.

## Conflicts of Interest

The authors declare no conflicts of interest.

## Supporting information


Supporting File 1

Supporting File 2

Supporting File 3

## Data Availability

The data that support the findings of this study are available from the corresponding author upon reasonable request. Individual Delphi responses are not publicly available in order to preserve participant anonymity. Aggregated data and Supporting materials are available from the corresponding author upon reasonable request.
