# Supplementary material for: Defining Core Evidence‐Based Practice Knowledge for Health Professionals: A Delphi Consensus Study
Source: J Eval Clin Pract. 2026 Oct 15;32(7):e70668. doi: 10.1111/jep.70668 (PMC13671091; doi:10.1111/jep.70668)
Supplement: Supplementary file 1 [file JEP-32-0-s001.pdf]

Preliminary EBP Knowledge Taxonomy Submitted to the First Delphi Round

The preliminary taxonomy was intended to describe the knowledge required to understand and engage with the Evidence-Based Practice (EBP) process.

The proposed knowledge domains were organised into dimensions reflecting the different stages of the EBP process.

Each dimension included several content areas. Together, they intended to represent the proposed content domain of EBP knowledge.

Dimension 1: Formulation of Clinical Questions Regarding Practice

This dimension refers to the process of formulating clear and specific clinical questions in order to address problems or uncertainties in practice. Health professionals should follow a systematic and structured approach to identify the need for information, translate it into a clear question, and classify it according to its type and objective.

This dimension comprises 3 content areas.

1.1 Translation from natural language to scientific language: Converting information and knowledge from the everyday language used in clinical practice into specialised terminology and structures suitable for posing a formal question.

1.2 Structuring the question: Knowledge of how to correctly structure and classify a clinical question according to context or discipline, study type, and the specific purpose being addressed.

1.3 Determining its objective: Identifying and defining the purpose or aim of a question, considering its clinical context, population, and intended objective.

Dimension 2: Scientific Literature Search

This dimension focuses on locating and selecting relevant information from scientific and technical literature sources. This process requires effective searching and the use of appropriate search terms.

This dimension comprises 3 content areas.

2.1 Electronic resources for evidence searching: Use of tools and technologies that facilitate access to and retrieval of relevant evidence, as well as guidance on formulating search strategies.

2.2 Search for external evidence: order of searching: Ranking and prioritising evidence sources according to their level of validity and degree of processing.

2.3 Search strategy: Planning and conducting a systematic search to maximise retrieval of relevant information and minimise the omission of pertinent evidence.

Dimension 3: Critical Appraisal of Results

This dimension refers to the critical evaluation and analysis of the studies and evidence identified, considering their validity, relevance, and applicability to the clinical context.

This dimension comprises 4 content areas.

3.1 Study types: Knowledge of the characteristics and designs of the different types of studies used in research.

3.2 Statistical interpretation of the study: Understanding and assessing the methodology of a scientific study by critically interpreting its design, variables, and, in quantitative studies, the statistical analysis of the data.

3.3 Critical appraisal tools: Knowledge of tools and approaches used to evaluate and critically analyse scientific literature.

3.4 Journal impact indicators: Knowledge of indicators used to assess a journal's influence and visibility within the academic community.

Dimension 4: Classification and Hierarchisation of Scientific Evidence

After individual appraisal of the identified studies, this dimension addresses the collective classification and ranking of evidence for its potential application in practice.

This dimension comprises 3 content areas.

4.1 Levels of evidence: Knowledge of hierarchical classifications used to assess and categorise the strength of evidence.

4.2 Quality of evidence: Knowledge of approaches used to assess the reliability, validity, and applicability of research findings.

4.3 Grades of recommendation: Knowledge of systems used to determine the degree of confidence in whether a practice should be applied, based on multiple factors.

Dimension 5: Implementation of Evidence and Knowledge Translation

This dimension refers to strategies used to adopt and integrate evidence-based health interventions, including staff training, adaptation of healthcare systems, and the development of support tools.

This dimension comprises 3 content areas.

5.1 Theoretical approaches to knowledge translation: Knowledge of theories, models, and conceptual frameworks intended to understand and facilitate the transfer of evidence into practice.

5.2 Implementation science resources: Understanding barriers and facilitators, together with resources that provide methodological guidance on implementation.

5.3 Implementation steps: Knowledge of the processes and stages that provide a structured guide for planning, carrying out, and evaluating evidence implementation in clinical practice.

Dimension 6: Evaluation of the EBP Process

This dimension refers to the systematic and continuous evaluation of the EBP process and of the implementation of interventions in clinical settings, with the aim of monitoring and improving quality and effectiveness.

This dimension comprises 4 content areas.

6.1 Recording of information and outcome measures: Knowledge of the appropriate storage, integrity, and availability of information required to evaluate outcomes.

6.2 Tools or instruments for analysing information: Knowledge of standardised tools or instruments used to analyse recorded information.

6.3 Indicators for evaluating implementation: Knowledge of frameworks used to classify indicators and objectively assess implementation success.

6.4 Sustainability of knowledge use: Knowledge of how to assess whether an innovation is maintained over time, considering changes in practice, leadership, organisational culture, training, and resources.

Dimension 7: Generation of New Scientific Evidence

This dimension refers to the generation of original information, data, and knowledge through research.

This dimension comprises 5 content areas.

7.1 Data collection techniques: Understanding the methods and instruments available for collecting research data.

7.2 Scientific writing: Knowledge and skills required to prepare clear, precise, and structured scientific documents for disseminating findings.

7.3 Publication standards for studies: Familiarity with reporting, editorial, and ethical requirements for publishing research.

7.4 Bibliographic references: Knowledge of citation styles and reference management software used to organise sources.

7.5 Ethical aspects of research: Knowledge of ethical principles guiding the design and conduct of research and safeguarding participants and data integrity.
