# Supplementary material for: Defining Core Evidence‐Based Practice Knowledge for Health Professionals: A Delphi Consensus Study
Source: J Eval Clin Pract. 2026 Oct 15;32(7):e70668. doi: 10.1111/jep.70668 (PMC13671091; doi:10.1111/jep.70668)
Supplement: Supplementary file 2 [file JEP-32-0-s002.pdf]

Final EBP Knowledge Taxonomy Following the Two-Round Delphi Study

The final taxonomy was established after two Delphi rounds and represents the content areas retained following expert evaluation.

The taxonomy is organised into five dimensions reflecting the principal stages of the Evidence-Based Practice (EBP) process.

Together, the dimensions and content areas define the proposed domain of core EBP knowledge.

Dimension 1: Formulation of a Clinical Question

This dimension refers to the process of formulating clear and specific clinical questions to address problems or uncertainties in practice. Health professionals should follow a systematic and structured approach to identify an information need and translate it into a clinically relevant question aligned with its type and purpose.

This dimension comprises 3 content areas.

1.1 Translation from natural language to scientific language: Converting information and knowledge from everyday clinical language into specialised terminology and structures suitable for posing a formal question.

1.2 Structure of the question: Knowledge of how to correctly structure a clinical question according to context or discipline, study type, and the type of question being posed.

1.3 Types of clinical questions: Identifying and defining the purpose or aim of a question, considering its clinical context, population, and objective.

Dimension 2: Scientific Literature Search

This dimension focuses on locating and selecting relevant information from scientific and technical sources using appropriate search strategies and terminology.

This dimension comprises 2 content areas.

2.1 Electronic resources and sources of evidence: Knowledge of the tools and technologies that facilitate the search for and access to relevant evidence, taking into account the degree of validity and level of processing of different sources.

2.2 Search strategy: Planning and conducting a systematic search, with knowledge of its components, to maximise retrieval of relevant information and minimise omission of pertinent evidence.

Dimension 3: Critical Appraisal of Results

This dimension encompasses the evaluation and analysis of selected studies and evidence, with emphasis on the validity, relevance, and applicability of findings in the clinical context. It includes consideration of study design, methodological and statistical rigour, potential biases, clinical interpretation, integration with previous knowledge, and dissemination of findings.

This dimension comprises 4 content areas.

3.1 Study designs: Knowledge of the characteristics and methodological aspects of the different types of studies.

3.2 Methodological appraisal: Critical interpretation of study design, variables, and potential biases and, in quantitative studies, statistical analysis.

3.3 Applicability of evidence: Understanding levels of evidence, strength of recommendations, and overall quality of evidence in order to judge clinical applicability.

3.4 Ethical aspects of research: Knowledge of ethical principles governing the conduct, management, use, and dissemination of research, considered as part of critical appraisal.

Dimension 4: Implementation of Evidence and Knowledge Translation

This dimension refers to strategies used to adopt and integrate evidence-based health interventions, including staff training, adaptation of healthcare systems, development of support tools, and monitoring of implementation.

This dimension comprises 2 content areas.

4.1 Implementation science resources: Understanding barriers and facilitators, together with resources from which methodological guidance on implementation can be obtained.

4.2 Implementation steps: Knowledge of the processes and stages that enable systematic planning, implementation, and evaluation of evidence use in clinical practice.

Dimension 5: Evaluation of the EBP Process

This dimension refers to the systematic and continuous evaluation of the EBP process and of the implementation of interventions in clinical settings, with the aim of monitoring and improving quality and effectiveness.

This dimension comprises 4 content areas.

5.1 Recording of information and outcome measures: Knowledge of the appropriate storage, integrity, and availability of data required to evaluate EBP, together with outcome measures relevant to each area of practice.

5.2 Tools or instruments for analysing information: Knowledge of the analysis of recorded information using standardised tools or instruments.

5.3 Indicators for evaluating implementation: Knowledge of frameworks used to classify indicators and objectively assess implementation success.

5.4 Sustainability of knowledge use: Knowledge of how to assess whether evidence or an evidence-based intervention is maintained over time, considering changes in practice, leadership, organisational culture, training, and available resources.
