# Supplementary material for: Defining Core Evidence‐Based Practice Knowledge for Health Professionals: A Delphi Consensus Study
Source: J Eval Clin Pract. 2026 Oct 15;32(7):e70668. doi: 10.1111/jep.70668 (PMC13671091; doi:10.1111/jep.70668)
Supplement: Supplementary file 3 [file JEP-32-0-s003.pdf]

Revised EBP Knowledge Taxonomy Submitted to the Second Delphi Round

The revised taxonomy was developed following the quantitative ratings and qualitative feedback obtained in the first Delphi round.

The proposed knowledge domains were organised into dimensions reflecting the different stages of the EBP process.

Each dimension included several content areas. Together, they intended to represent the revised content domain of EBP knowledge.

Dimension 1: Formulation of a Clinical Question

This dimension refers to the process of formulating clear and specific clinical questions to address problems or uncertainties in practice. Health professionals should follow a systematic and structured approach to identify an information need and translate it into a clinically relevant question aligned with its type and purpose.

This dimension comprises 3 content areas.

1.1 Translation from natural language to scientific language: Converting information and knowledge from everyday clinical language into specialised terminology and structures suitable for posing a formal question.

1.2 Structure of the question: Knowledge of how to correctly structure a clinical question according to context or discipline, study type, and the type of question being posed.

1.3 Types of clinical questions: Identifying and defining the purpose or aim of a question, considering its clinical context, population, and objective.

Dimension 2: Scientific Literature Search

This dimension focuses on locating and selecting relevant information from scientific and technical literature sources, requiring appropriate search strategies and terminology.

This dimension comprises 2 content areas.

2.1 Electronic resources and sources of evidence: Use of tools and technologies that facilitate the search for and access to relevant evidence, taking into account the degree of validity and level of processing of different sources.

2.2 Search strategy: Planning and conducting a systematic search, with knowledge of its components, to maximise retrieval of relevant information and minimise omission of pertinent evidence.

Dimension 3: Critical Appraisal of Results

This dimension refers to the critical evaluation and analysis of selected studies and evidence, considering validity, relevance, applicability, integration with previous knowledge, and communication of findings.

This dimension comprises 5 content areas.

3.1 Study types: Knowledge of the characteristics and designs of the different types of studies.

3.2 Methodological appraisal: Understanding and assessing the methodology of a scientific study by critically interpreting its design, variables, and, in quantitative studies, the statistical analysis of the data.

3.3 Applicability of evidence: Knowledge of levels of evidence, grades of recommendation, and overall quality of evidence in order to judge the applicability of research findings to clinical practice.

3.4 Ethical aspects of research: Knowledge of the principles that guide research and should be considered when critically appraising evidence.

3.5 Bibliographic references: Knowledge of citation standards and reference management tools used to organise sources.

Dimension 4: Implementation of Evidence and Knowledge Translation

This dimension refers to strategies used to adopt and integrate evidence-based health interventions, including staff training, adaptation of healthcare systems, development of support tools, and improvement of patient care and outcomes.

This dimension comprises 2 content areas.

4.1 Implementation science resources: Understanding barriers and facilitators, together with resources from which methodological guidance on implementation can be obtained.

4.2 Implementation steps: Knowledge of the processes and stages that enable systematic planning, implementation, and evaluation of evidence use in clinical practice.

Dimension 5: Evaluation of the EBP Process

This dimension refers to the systematic and continuous evaluation of the EBP process and of the implementation of interventions in clinical settings, with the aim of monitoring and improving quality and effectiveness.

This dimension comprises 4 content areas.

5.1 Recording of information and outcome measures: Knowledge of the appropriate storage, integrity, and availability of data required to evaluate EBP, together with outcome measures relevant to each area of practice.

5.2 Tools or instruments for analysing information: Knowledge of the analysis of recorded information using standardised tools or instruments.

5.3 Indicators for evaluating implementation: Knowledge of frameworks used to classify indicators and objectively assess the success of an implementation project.

5.4 Sustainability of knowledge use: Knowledge of how to assess whether evidence or evidence-based interventions are maintained over time, considering leadership, organisational culture, training, and available resources.
